# Predictors of response after single session interventions for emotional distress: using enhanced psychoeducation in crisis situations

**DOI:** 10.47626/1516-4446-2024-3749

**Published:** 2024-11-25

**Authors:** Ana Luiza da Silva Ache, Bruno Braga Montezano, Bruno Paz Mosqueiro, Marco Antonio Caldieraro, Lucas Spanemberg, Giovanni Abrahão Salum, Marcelo P. Fleck

**Affiliations:** 1 Programa de Pós-Graduação em Psiquiatria e Ciências do Comportamento, Departamento de Psiquiatria, Universidade Federal do Rio Grande do Sul, Porto Alegre, RS, Brazil; 2 Hospital de Clínicas de Porto Alegre, Porto Alegre, RS, Brazil; 3 Programa de Pós-Graduação em Medicina e Ciências da Saúde, Escola de Medicina, Pontifícia Universidade Católica do Rio Grande do Sul, Porto Alegre, RS, Brazil; 4 Programa de Pós-Graduação em Ciências Criminais, Escola de Direito, Pontifícia Universidade Católica do Rio Grande do Sul, Porto Alegre, RS, Brazil

**Keywords:** Mental health, pandemics, COVID-19, psychological distress

## Abstract

**Objective:**

Single-session interventions are an effective strategy for reducing emotional distress. Enhanced psychoeducation, which includes empathic listening, risk stratification, symptom monitoring, and habit modification is particularly suitable for single-session interventions. We investigated predictors of response to an online enhanced psychoeducation intervention among essential service professionals during the COVID-19 pandemic in Brazil.

**Methods:**

The TelePSI Project, financed by the Brazilian Ministry of Health, was a nationwide initiative that served more than 3,300 individuals in various psychotherapeutic modalities. Data were collected from April 2020 to December 2021. We included all participants with high levels of emotional distress who received single-session interventions. The final sample included 460 individuals (89.1% women, 81.1% health professionals). After 1 month, 300 participants were reassessed.

**Results:**

Overuse of social media, use of social networks to contact family and friends, playing video games, smoking, drinking alcohol, and spending time with pets were associated with less improvement in symptoms, whereas playing an instrument and previous psychological treatment were associated with greater symptom improvement. This highlights the impact of lifestyle factors on the efficacy of single-session interventions.

**Conclusion:**

These results underscore the importance of considering individual lifestyle factors when implementing single-session interventions and contribute to a growing body of evidence that supports tailored application of psychoeducational strategies in mental health interventions, particularly in high-stress environments.

## Introduction

The COVID-19 pandemic had a major impact on the world’s emotional health, and knowledge is limited about the efficacy of traditional interventions in this context.^1^,^2^ One such intervention, psychoeducation, has been employed to alleviate emotional distress among essential workers.^3^,^4^ Psychoeducation involves providing patients with information about their symptoms and treatment, fostering behavioral changes and actively engaging patients in their treatment.^3^-^7^ This approach can encourage lifestyle changes, such as healthier eating, exercise, and sleep hygiene, leading to enhanced quality of life. Psychoeducation also effectively reduces symptoms and improves prognosis in those with serious health conditions or who have been exposed to trauma, even after a single-session intervention.^8^-^11^ A systematic review on single-session interventions found that they were effective in ameliorating psychiatric disorders, particularly depression.^12^ Thus, single-session interventions show promise as a psychological care strategy for emotional problems.^10^,^12^-^15^

The TelePSI Project, which was initiated by the Hospital de Clínicas de Porto Alegre in collaboration with the Brazilian Ministry of Health, aimed to provide online mental health care to healthcare professionals working in essential services during the COVID-19 pandemic.^16^-^18^ The project’s main objective was to compare three psychosocial interventions (ultra-brief cognitive-behavior psychotherapy, ultra-brief interpersonal psychotherapy, and a single session of enhanced psychoeducation [EP]). EP, an innovative strategy proposed by TelePSI, addresses emotional symptoms identified in an initial assessment with the participant, suggesting lifestyle changes based on individual risk and protective factors for physical, emotional, psychological, and well-being. This enhanced form of psychoeducation is intended to be both informative and therapeutic. Participants who received EP with support videos also received videos developed by TelePSI for 4 weeks.^16^,^19^

The main conclusion of the TelePSI Project was that, surprisingly, a single session of EP performed equally as well as the other interventions, although it was briefer and simpler.^20^ However, almost all participants improved to some degree after a single session of EP. Since the factors involved in these significant results were unclear, this study was an exploration of EP-related factors that lead to increased improvement in an effort to determine the profile of patients who most benefit most from this intervention.

## Methods

The study sample was drawn from that of the general TelePSI Project, in which participants who underwent EP significantly improved, including symptom remission that was sustained over time.^20^ The present study will not focus on who did or did not improve, but rather on factors that contributed to a better response.

Follow-up assessments, including the Patient-Reported Outcomes Measurement Information System (PROMIS) scales and other lifestyle, COVID exposure, and risk assessments were applied online via self-report questionnaires at 1, 3, and 6 months. There was significant improvement at 1 month, which was maintained in the 3- and 6-month reassessments. Thus, the 1-month data will be used to identify factors that may be involved in greater improvement. Additional details about the project can be found in the main article.^20^-^22^

Patients were classified into two groups based on severity and symptomatology: high risk (T scores > 70 points in any of the emotional stress scales) and low risk (T-score < 70 points on all of the emotional stress scales). Low-risk participants were randomized to two groups: EP with and without support videos. High-risk participants were randomized to EP, interpersonal therapy, or cognitive behavioral therapy.

### Study sample

The TelePSI Project offered free mental health interventions to medical doctors, biomedical doctors, nurses, nursing technicians, physical therapists, speech therapists, nutritionists, pharmacists, health students, essential services professionals, and teachers. The project provided various types of care and collected data from April 2020 to December 2021. This study only analyzed data from high-risk participants randomized to the EP intervention with video reinforcement. Exclusion criteria consisted of inability to answer questionnaires and indication for immediate evaluation in an urgent/emergency service due to the risk of suicide/aggression toward others.

### Intervention

EP with reinforcement videos consisted of a single, structured psychoeducation session lasting approximately 50 to 60 minutes, followed by 4 weeks of forwarded videos. The session occurred online via Google Meet, with the therapist providing empathetic listening and identifying the patient’s mental health needs and areas of greatest concern. During the session, symptoms, risk factors, and protective factors for health were addressed, and lifestyle changes were encouraged. Patients were advised to seek face-to-face or emergency care in their locality in case of worsening or acute risk situations. After the session, short videos developed by the project team were sent to the participants. These videos focused on 16 mental health topics, including how to protect oneself from COVID-19 infection, fear of contagion, normal vs. excessive anxiety, normal sadness vs. depression, anger vs. irritability, burnout, stress and acute reaction to stress, sleep hygiene, healthy eating and mental health, exercise and mental health, excessive consumption of alcohol and drugs, excessive exposure to the news, excessive use of social networks, caring for children, caring for older adults, and social support.^19^ The therapist selected the eight most relevant videos for each patient and sent them through WhatsApp messages twice a week. The videos were based on various psychotherapeutic approaches and emphasized psychoeducation and lifestyle change to enhance the participants’ quality of life.

### Measures

The PROMIS Scales of Depression, Anxiety, Anger, Satisfaction with Life, and Sleep were utilized to assess symptom severity. PROMIS developed self-report measures for adults to evaluate functioning, symptoms, behaviors, and feelings. These patient assessment measures were designed for application at the initial patient interview and to monitor treatment progress, using baseline symptom status and patient-reported outcome information, as well as instrument-based severity assessment.^23^

PROMIS Depression Short Form is an eight-item self-report scale to assess health status for depression. Participants were asked to rate their experience for each item in the past 7 days on a five-point scale from 1 (never) to 5 (always).^23^

PROMIS Anxiety Short Form is an eight-item self-report scale to assess health status for anxiety. Participants were asked to rate their experience for each item in the past 7 days on a five-point scale from 1 (never) to 5 (always).^23^

PROMIS Anger Short Form is a five-item self-report scale to assess angry mood (irritability, frustration), negative social cognitions (interpersonal sensitivity, envy, disagreeableness), and efforts to control anger on a five-point scale from 1 (never) to 5 (always).^23^,^24^

PROMIS General Life Satisfaction Short Form is a five-item self-report scale to assess cognitive evaluation of life experiences and life satisfaction on a five-point scale from 1 (never) to 5 (always).^23^,^24^

PROMIS Sleep Disturbance-Short Form is an eight-item self-report scale to evaluate sleep problems, sleep quality, sleep depth, and restoration associated with sleep on a five-point scale from 1 (never) to 5 (always).^24^,^25^

### Study design

A theoretical model was developed for the TelePSI Project based on a biopsychosocial understanding of factors that could influence mental health. Independent variables associated with significant improvement were analyzed. The variables were collected at the baseline and 1-month post-treatment assessment. Baseline variables included age, sex, employment as a health professional, baseline symptoms, COVID exposure, prior mental health treatment, medication use, and suicidal ideation. Potential protective factors (e.g., watching videos on YouTube, meditating, praying, writing, spending time with friends and family online, exercise, playing a musical instrument, listening to music, spending time with pets, playing video games, reading books, artistic activities, playing board games, and healthy eating) and potential risk factors (e.g., consuming excessive carbohydrates, drinking soda, drinking alcohol, smoking, using marijuana, using other drugs, self-medicating, excessive television watching, arguing on social media, assiduous Internet news consumption, and poor sleep) were also assessed. At the 1-month follow-up assessment, data on variables such as the number of videos watched, work absences, perceived improvement, and treatment satisfaction were collected.

### Statistical analysis

Categorical variables were described using absolute and relative frequencies, while continuous variables were described as mean and standard deviation, as well as median and range (minimum and maximum). The Wilcoxon signed-rank test was used to assess symptom improvement using PROMIS scores from baseline to follow-up for outcomes where score changes were not normally distributed; otherwise, a paired t-test was used.

Linear mixed models with restricted maximum likelihood criterion were constructed to assess the effect of time and independent baseline variables on symptom trajectory for the five different outcomes (depression, anxiety, irritability, life satisfaction, and sleep). A separate linear mixed model was constructed for each combination of dependent and independent variables.

All continuous variables were standardized using z-scores to enhance interpretability. A significance level of 0.05 was used in all models. The linear mixed model results for the effects of risk and protective factors were presented separately in models where baseline scores differed significantly (significant fixed effect regression coefficients).

All analyses were performed using scripts written in R 4.3.2. The linear mixed model was built using lme4 1.1. The dplyr 1.1.0, broom 1.0.3, tidyr 1.3.0, purrr 1.0.1, and ggplot2 3.4.0 packages were used for data analysis routines.

## Results

Due to the sample’s heterogeneity, baseline scores differed for some independent variables. Thus, the results were divided into two categories: participants with similar baseline scores and participants with different baseline scores on the PROMIS severity scales.

The final sample consisted of 460 participants, predominantly women (89.1%) and health professionals (81.1%), with an average age of 35.6 (SD, 9.3) years. One month later, 300 participants were reassessed. Table 1 provides a summary of the sample’s demographic and clinical characteristics. At 1 month, EP significantly improved participant symptoms (irritability, depression, anxiety) and was also associated with better sleep and life satisfaction compared to baseline, as was reported in the main study.^20^ In the present study, we focus on factors associated with improvement after EP.

### Baseline similarities

Participants began with similar baseline scores for the analyzed variables. For depression scores, older individuals and people who spent time with pets showed less improvement after a month. For the anxiety outcome, using social networks for fun or conflict, spending time with pets, playing video games, alcohol use, and smoking at baseline were associated with less improvement.

For irritability, factors associated with less improvement included time spent with pets, playing video games, and smoking. For sleep quality scores, playing video games was associated with less improvement. Receiving some form of mental health treatment at baseline was associated with greater improvement than not being in treatment. Younger participants and those who played an instrument had greater life satisfaction.

The main results of the linear mixed models are shown in Table 2. We outline the factors associated with greater or lesser variation in PROMIS scores after 1 month. To illustrate, we will consider one variable in the depression outcome: time spent with pets. After 1 month, both groups improved; however, those who did not spend time with their pets at baseline had greater improvement than those who did. Participants who did not spend time with their pets had a more substantial decrease (2.17 points) in the final PROMIS depression score, indicating they were less symptomatic participants who spent time with their pet.

### Baseline differences

**Figure 1:**
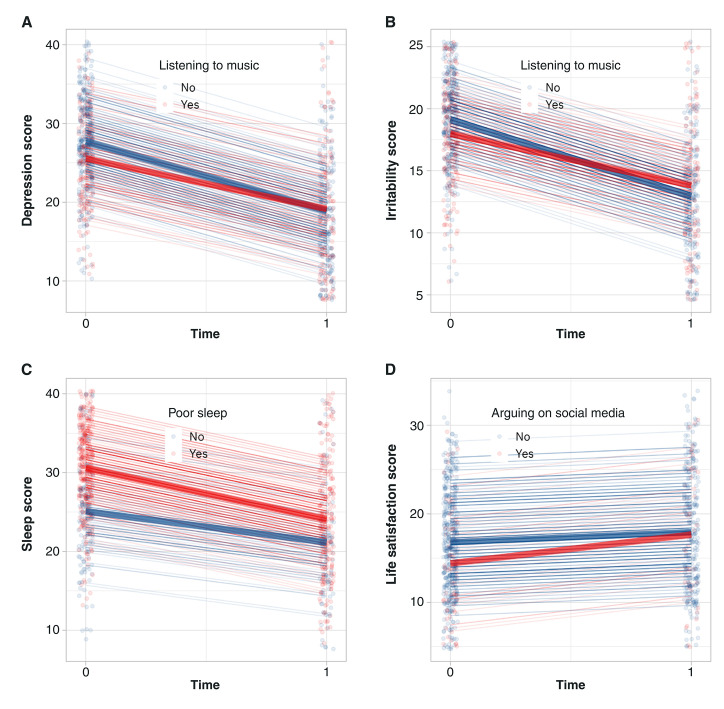
Linear mixed model interaction for independent variables with significant baseline differences and significant estimates of interaction between factor and time, considering α = 0.05 (n=300). The graphs correspond to the models presented in Table 3: (A) the effects of listening to music on depression score, (B) the effects of listening to music on irritability score, (C) the effects of poor sleep on sleep score, and (D) the effects of arguing/“getting stressed” on social media on life satisfaction score. Red color means “Yes” (positive answer) and blue means “No” (negative answer) for all presented subgraphs.

Despite randomization, not all individuals had the same level of symptoms in all areas, starting with different baseline scores. Figure 1 and Table 3 show variables that could not be considered predictors of different outcomes because the differences were based on varying baseline scores. Table 3 shows the variables for which individuals began at different symptom severity levels at baseline. For example, patients who listened to music had a β (x1) 2.14 points lower than those who did not. The group that listened to music improved by around 2.4 fewer points β (time × x1). This means that these individuals had fewer baseline depression symptoms but improved less at the 1-month follow-up, although those who did not listen to music at the beginning of the study had an improvement delta of 2.43 points, indicating a greater magnitude of improvement. The two groups had very similar scores during follow-up, but the group that already listened to music at baseline, being less symptomatic, had less room for improvement (Figure 1).Those who listened to music showed improvement in irritability and depression, but further analysis revealed that these patients had fewer symptoms at baseline.

## Discussion

Participants who overused social media, used social networks to contact family and friends, played video games, smoked tobacco, drank alcohol, and spent time with pets had less improvement. Advanced age was associated with less improvement, i.e., the older the age, the smaller the range of improvement. However, participants who were receiving some psychological treatment at baseline had greater improvement in sleep quality, and participants who played an instrument at baseline had greater improvement in life satisfaction.

Both the overuse of social media and the use of social networks to contact family and friends were associated with less improvement in anxiety symptoms. Internet and social media use significantly increased during the pandemic due to social isolation and lockdown measures, leading to fear of and misinformation about the disease.^26^ According to Bendau et al.,^27^ the media type and exposure time affect negative psychological outcomes, resulting in significantly higher anxiety levels during the pandemic. Overuse of social media has also been associated with a significant increase in symptoms, particularly anxiety, as demonstrated Dumer et al.^28^ Playing video games proved to be a negative factor for anxiety, irritability, and poor sleep quality, and individuals in this group showed less improvement after the EP intervention. Although video games, especially those involving interpersonal contact, can alleviate stress, anxiety, depression, and loneliness during lockdown, for individuals with risk factors, playing video games had harmful effects during pandemic.^29^ While technology is also associated with mental health benefits, this depends on the way it is used and its objectives.^30^,^31^ It was by design that TelePSI is an online tool and videos were also sent via WhatsApp, because one objective of the EP intervention was to promote a healthier relationship with social media, technology, and avoid information overconsumption, which was frequently observed during the pandemic.

Tobacco and alcohol use were also associated with less improvement in irritability and anxiety symptoms, respectively. Psychoactive substance use is an important risk factor for mental disorders.^32^ We did not investigate whether participants who reported drug use were also diagnosed with substance use disorder, nor did we obtain detailed information about their consumption patterns or the impact of substance use on their behavior and functionality. Substance use could be identified in the questionnaires or even during the session, and there was a specific EP video on substance use, including alcohol and tobacco, to increase patient awareness. However, it is reasonable to assume that treatment for substance use disorder is too complex to be addressed in a single psychoeducational intervention.

Contrary to what we might assume regarding pets and mental health, people who reported spending time with a pet during the pandemic had less improvement than those who did not. A meta-analysis by Martins found relatively little or no benefit from pets during the pandemic.^33^ Some studies have identified an increased burden among pet owners during the pandemic that contributed to lower quality of life.^34^,^35^ As in the present study, these individuals had less improvement in depression, anxiety, and irritability symptoms, which may be because pets do not replace the interpersonal exchanges of family and work relationships, as demonstrated by Ogata et al.^36^ Our sample predominantly consisted of healthcare professionals on the frontlines against COVID-19, who were already under great pressure. It is possible that pet ownership may involve increased care demands and responsibilities during downtime at home.

Older participants experienced less improvement from the intervention. This may be because digital interventions and interaction are more appealing to younger people. At the start of the pandemic, many participants, especially older adults, were unfamiliar with digital interventions. Despite the availability and potential benefits of health technologies, accessing them may be more difficult for older people, and they may have had previous negative experiences with digital tools. These individuals would need more training and education on how to use technology to their advantage, including longer adaptation time.^37^ Another hypothesis is that age was an important risk factor for severe COVID-19, which could also have increased fear and emotional symptoms during the pandemic. Moreover, a large part of our sample was highly exposed to the virus due to their profession, increasing the possibility of infection. Such reasons could explain why age was associated with less improvement.

Concerning the life satisfaction outcome, the higher the score, the greater the satisfaction. Participants who played an instrument had a greater improvement in life satisfaction scores at 1 month. We hypothesize that musicians have a hobby that could be pursued at home, which could have served as a coping strategy during lockdown. Music performance has been identified as a positive factor for mental health during the pandemic,^38^ a finding that our results support, since playing an instrument was associated with better improvement after EP. A study found that individuals who play instruments had lower depression scores during the pandemic than those who only sang or played no instrument.^39^ Contact with art during the pandemic has been described as an important way to connect despite social distancing, and it was found to have a positive effect on sleep quality.^40^

As shown in Figure 1 and Table 3, participants who listened to music had fewer baseline symptoms, suggesting they had less room for improvement. It may be that frequently listening to music prior to the intervention served as a protective factor/coping mechanism. Consequently, a lesser degree of symptom improvement would occur due to their pre-existing coping strategies.

This study has some limitations. First, many participants were lost to follow-up. Despite attempted contact via mobile phone, 160 participants did not respond to the follow-up questionnaires. Secondly, the assessments were conducted at different times during the pandemic, characterized by increasing waves of cases, which could have affected symptom severity. Data relating to lifestyle changes over one month were not analyzed and will be explored in other TelePSI studies.

There was an exponential increase in digital interventions during the pandemic.^41^ Most of these digital health tools have not been clinically validated.^42^ It is important not only to study this type of intervention, but to develop a theoretical basis for them. We hypothesize that EP could also be useful not only in adverse situations, such as pandemics, but also in more simple and common circumstances. EP not only uses passive strategies for psychoeducation but encourages active involvement.^43^ Sending videos tailored to the participant needs, an innovation of TelePSI, can extend the effect of traditional psychoeducation beyond the single-session intervention, since it keeps participants engaged and connected with the therapist for 4 weeks after the consultation. Although interest in health technologies has increased steadily in the last decade and exponentially during the pandemic, there is still much to be explored in this field.

Online EP with support videos, as proposed by TelePSI, appears to be an effective intervention strategy for healthcare professionals with symptoms of anxiety, depression, and irritability. Although a significant proportion of the participants improved, some variables were associated with less improvement, such as spending time with pets, tobacco use, alcohol use, and playing video games. In this study, we set out to analyze variables associated with participant improvement because, by evaluating these factors, we can better understand this new model of psychoeducation, which represents a promising alternative for individuals during crises such as the COVID-19 pandemic. We believe that new psychotherapeutic strategies can thus be recommended in a more personalized way. EP is a low-cost and effective approach for symptom improvement that can be offered for remote locations, especially in large territories with limited transportation infrastructure. More research is needed to compare the intervention’s effectiveness with other strategies. Furthermore, the intervention should be explored beyond the context of the pandemic and social isolation. Further research on TelePSI is forthcoming, including a comparison of intervention arms.

## Disclosure

The authors report no conflicts of interest.

## Figures and Tables

**Table 1:** Descriptive statistics of the participants’ sociodemographic and clinical variables (n=460)

Variable	Overall
Sex	
Female	410 (89.1)
Age	
Mean (SD)	35.60 (9.29)
Median (Min, Max)	35.00 (19.00, 65.00)
Health professional	
Yes	373 (81.1)
Number of videos watched (follow-up)†	
Mean (SD)	5.81 (2.47)
Median (Min, Max)	6.00 (1.00, 11.00)
Number of work absences†	
Mean (SD)	2.32 (5.89)
Median (Min, Max)	0.00 (0.00, 30.00)
Perceived improvement†	
Much worse	3 (0.7)
Moderately worse	3 (0.7)
Slightly worse	7 (1.5)
About the same	53 (11.5)
Slightly better	90 (19.6)
Moderately better	85 (18.5)
Much better	59 (12.8)
Missing	130 (28)

Data presented as n (%), unless otherwise specified.

Max = maximum value; Min = minimum value.

† Missing about 35%.

**Table 2:** Lifestyle factors associated with significant difference in improvement over time in each outcome between groups,† α = 0.05 (n=300)

Independent variable	β (time × x_1_)‡	SE	p-value (time × x_1_)
Depression			
Age	-0.771	0.383	0.045
Spending time with one’s pet(s)	-2.17	0.882	0.014
Anxiety			
Age	-1.97	0.379	0.002
Having fun on social media with friends and family	-1.97	0.929	0.034
Spending time with one’s pet(s)	-1.97	0.875	0.025
Playing video games	-3.48	1.750	0.047
Drinking alcohol	-1.89	0.922	0.041
Fighting or “getting stressed” on social media when in contact with friends or family	-2.71	1.09	0.013
Irritability			
Spending time with one’s pet(s)	-1.380	0.636	0.030
Playing video games	-2.980	1.270	0.019
Smoking	-2.660	1.060	0.012
Sleep			
Playing video games	-5.87	1.77	0.001
Receiving treatment for emotional or behavioral problems?	1.69	0.803	0.036
Life satisfaction			
Age	0.708	0.335	0.035
Playing an instrument	-4.63	2.27	0.042

SE = standard error.

† Minus sign = less improvement; plus sign = more improvement.

‡ Change in Patient-Reported Outcomes Measurement Information System after 1 month of follow-up.

**Table 3:** Linear mixed model results for independent variables with significant baseline differences and significant estimates of interaction between factor and time, considering α = 0.05 (n=300)

Independent variable	β (x_1_)	SE	p-value (x_1_)	β (time × x_1_)	SE	p-value (time × x_1_)
Depression						
Listening to music	-2.14	0.645	< 0.001	-2.43	0.767	0.002
Irritability						
Listening to music	-1.18	0.419	0.005	-2.02	0.551	<0.001
Sleep						
Poor sleep (quantity and/or quality)	5.45	0.674	< 0.001	2.56	0.810	0.002
Life satisfaction						
Fighting/“getting stressed” on social media when in contact with friends or family	-2.37	0.857	0.006	-2.11	0.961	0.029

SE = standard error.
